# Emergency treatment of a snake bite: Pearls from literature

**DOI:** 10.4103/0974-2700.43190

**Published:** 2008

**Authors:** Syed Moied Ahmed, Mohib Ahmed, Abu Nadeem, Jyotsna Mahajan, Adarash Choudhary, Jyotishka Pal

**Affiliations:** Department of Anesthesiology and Critical Care, JN Medical College, Aligarh Muslim University, India

**Keywords:** Anti snake venom, global issue, snakebite

## Abstract

Snake bite is a well-known occupational hazard amongst farmers, plantation workers, and other outdoor workers and results in much morbidity and mortality throughout the world. This occupational hazard is no more an issue restricted to a particular part of the world; it has become a global issue. Accurate statistics of the incidence of snakebite and its morbidity and mortality throughout the world does not exist; however, it is certain to be higher than what is reported. This is because even today most of the victims initially approach traditional healers for treatment and many are not even registered in the hospital. Hence, registering such patients is an important goal if we are to have accurate statistics and reduce the morbidity and mortality due to snakebite. World Health Organization/South East Asian Region Organisation (WHO/SEARO) has published guidelines, specific for the South East Asian region, for the clinical management of snakebites. The same guidelines may be applied for managing snakebite patients in other parts of the world also, since no other professional body has come up with any other evidence-based guidelines. In this article we highlight the incidence and clinical features of different types of snakebite and the management guidelines as per the WHO/SEARO recommendation.

Venomous animals account for much morbidity and mortality. Worldwide, it is estimated that more than 5 million persons per year are bitten by snakes, out of which approximately 100,000 develop severe sequelae.[1,2] The actual figure may be much more since in India alone the mortality is suggested to be around 30,000.[3] Out of the available yearly statistics, the United States alone had 50,000 cases of bites, of which 7000 were by venomous snakes. Interestingly, of those 7,000 snakebite victims, 1,200 did not seek medical attention and yet they fully recovered. In all, there were 15 fatalities and thus the chance of survival is 499 out of 500. According to an epidemiological survey among 19,000 individuals living in 26 villages in Burdwan district (West Bengal), there was an annual incidence of snakebite of 0.16% and a mortality rate of 0.016% per year.[4] Maharashtra reports an incidence of 70 bites per 100,000 population and a mortality of 2.4 per 100,000 persons per year.[5] Indian states with high incidence of snakebites cases are Tamil Nadu, West Bengal, Maharashtra, Uttar Pradesh, and Kerala.[6] In Myanmar (Burma) Russell's vipers are responsible for 90% of cases. In 1991, there were 14,000 bites with 1,000 deaths and in 1997, 8,000 bites with 500 deaths. Under-reporting is estimated at 12%.[7] In Bangladesh, a survey of 10% of the country in 1988–1989 revealed 764 bites with 168 deaths over the 1-year period. Cobra bites (34% of all bites) had a case fatality of 40%. In Vietnam there are an estimated 30,000 bites per year. Among 430 rubber plantation workers bitten by Malayan pit vipers between 1993 and 1998, the case fatality was 22%, but only a minority had received antivenom treatment. Fishermen are still occasionally bitten by sea snakes but rarely reach hospitals alive. Pakistan has an estimated 20,000 snakebite deaths each year.

In Nepal there is an estimated 20,000 snakebites each year, with about 200 deaths in hospitals, mainly in the Terai region. One survey suggested as many as 1,000 deaths per year. Among 16 fatalities recorded at one rural clinic during a monsoon season, 15 had died on their way to seek medical care.[8]

## EPIDEMIOLOGY

### Agent

Snakes are distributed throughout most of the earth's surface with some exceptions such as the Arctic, Antarctic, and many small islands. Snakes are poikilothermic carnivorous reptiles that have evolved the venomous apparatus for the purpose of procurement of food.[9] To a large extent the manifestation of snakebite depends upon the species of snake, and therefore identification of the type of snake is important. At times the bite mark might not be visible (e.g., in the case of krait). The killed snake brought as evidence helps in identification of snake, in which case species-specific monovalent Anti snake venom (ASV) can be administered. The clinical manifestations of the patient may not correlate with the species of snake brought as evidence. It is therefore advantageous to know the appearance of the snake so as to recognize the species.

The three major families of venomous snakes are the Elapidae, the Viperidae, and the Hydrophidae.

**Elapidae** (cobra, king cobra, krait, and coral snake): These snakes have heads that are of about the same width as their necks. The head is covered with large scales but lack laureal shields. Their pupils are round and they are oviparous. These snakes have grooved fangs that are short, fixed, and covered by mucous membrane. They, therefore, cannot bite through clothes and usually deliver only a sublethal dose.

**Viperidae** (vipers): The head of a viper is triangular, wider than the neck, and has laureal shields. They have vertically elliptical pupils and are ovi-viviparous. Their fangs are long, movable, and canalized like hypodermic needles. They are further subdivided into pit viper and pitless viper subfamilies. The Crotalinae (pit vipers) have a special sense organ, the pit organ, to detect their warm-blooded prey. This is situated between the nostril and the eye. The rattlesnake belongs to the pit viper subfamily, while the Russell's viper and the saw-scaled viper belong to the pitless viper subfamily.

**Hydrophidae** (sea snake): Sea snakes are found in the vicinity of the seacoast. They have a small head and a flattened tail that helps them swim. Though venomous, they seldom bite.

In India, more than 200 species of snakes have been identified but only 52 are poisonous; the common krait (Bungarus caeruleus), Indian cobra (Naja naja), Russell's viper (Daboia russelii), and saw-scaled viper (Echis carinatus) are the most poisonous (“the big four”).[10,11] In the Indian setting, almost two-thirds of bites are attributed to saw-scaled vipers, about one-fourth to Russell's viper, and only a small proportion to cobras and kraits.[12,13]

### Host and environmental factors

Thorough statistical analysis of snakebite is difficult and the available data is not always complete because of the varied distribution (and because most bites occur in remote villages). Snakebite may be termed an occupational disease, as farmers, plantation workers, herdsmen, hunters, or workers on development sites are mostly affected.[14] Snakebites show a classical seasonal variation, being more common in summers and in the rainy season, when it is associated with agricultural activities.[3] The majority of snakes do not bite without provocation; most bites are inflicted when the snakes are inadvertently trodden upon. Males are bitten almost twice as often as females,[15] with the majority of the bites being on the lower extremities.[16–18] Fortunately, 50% of bites by venomous snakes are “dry bites” that result in negligible envenomation. The percentage of dry bites ranges from 10–80% for various poisonous snakes.[19]

## SNAKE VENOM

### Chemical composition

The normal function of snake venom is to immobilize the prey and to assist in digestion. The toxic component of snake venom can be classified into four broad categories: enzymes, polypeptides, glycoproteins, and compounds of low molecular weight.[9] They can also be classified as protein (90–95%) and nonprotein (5–10%) compounds.[9] Table 1 provides the chemical composition of snake venom.

**Table 1:** Compounds present in snake venom

Enzymes	Phospholipase A_2_ (lecithinase), 5^¢-nucleotidase^, collagenase, L-amino acid oxidase, proteinases,hyaluronidase, acetylcholine esterase*, phospholipase B*, endopeptidase†, kininogenase†, Factor X†, prothrombin activating enzyme†
Nonenzyme polypeptides	Polysynaptic (a) neurotoxin (α- bungarotoxin and cobrotoxin), presynaptic (b) neurotoxin (β- bungarotoxin, crotoxin, and taipoxin) cardiotoxin, crotamine
Peptides	Pyroglutamylpeptide
Nucleosides	Adenosine, guanosine, inosine
Lipids	Phospholipids, cholesterol
Amines	Histamine, serotonin, spermin
Metals	Copper, zinc, sodium, magnesium

* In Elapids

† In Vipers

### Toxic effects of snake venom

The toxic effect of snake venom results from both the protein and the nonprotein component. It is further complicated by the inflammatory response of the victim's body.

Phospholipase A2 is present in the venom of all families of poisonous snakes and is the enzyme that has been most widely studied. Phospholipase A2 inhibits electron transfer at cytochrome C level and renders mitochondrial-bound enzymes soluble. It damages red blood cells, leukocytes, platelets, skeletal muscle, vascular endothelium, peripheral nerve endings, and the myoneural junction.[9]

Hyaluronidase helps spread of venom through tissues, and proteolytic enzymes are responsible for the local edema, blistering, and necrosis.[3]

α- Neurotoxins bind to acetylcholine receptors at the motor end-plate, whereas β- neurotoxins first cause release of acetylcholine at the nerve endings at the myoneural junction and then damage the endings, preventing further release of transmitter.[3] All this leads to a flaccid paralysis of the victim.

Polypeptides, being smaller molecules, are rapidly absorbed into the systemic circulation and cause systemic toxicity in vessel-rich organs (e.g., heart, lung, kidneys, etc.) as well as at pre- and postsynaptic membranes.

## CLINICAL FEATURES OF SNAKEBITE

Some people who are bitten by snakes (or suspect or imagine that they have been bitten) may develop quite striking symptoms and signs, even when no venom has been injected. This results from an understandable fear of the consequences of a real venomous bite. Anxious people may hyperventilate so that they develop pins-and-needles sensation in the extremities, spasm of their hands and feet, and dizziness. Others may develop vasovagal shock after the bite or suspected bite, with faintness and collapse with profound slowing of the heart. Others may become highly agitated and irrational and may manifest a wide range of misleading symptoms.[14]

The clinical presentation of a snakebite victim varies with the age and size of the patient, the species of snake, the number and location of the bites, and the quantity and toxicity of the venom.

Morbidity and mortality depends on the age and size of victim (children receive larger envenomation relative to body size) as well as comorbid conditions (elderly patients succumb more easily to snake venom).[9] Other factors affecting severity and outcome are listed in Table 2. Factors not contributing to outcome are size of the snake and time of bite (day/night).[14]

**Table 2:** Factors contributing to severity and outcome in snakebite

Factor	Effect on outcome
Size of victim	Bigger the size, good is the outcome due to less amount of toxin per kg of body weight
Comorbidity	Predisposes to harmful effect of snake venom
Part bitten	Patients bitten on the trunk, face, and directly into bloodstream have a worse prognosis
Exercise	Exertion following snake bite has poor outcome due to enhanced systemic absorption of toxin
Individual sensitivity	Sensitivity of individual to venom modifies the clinical picture
Bite characteristics	Bite number; depth of bite; dry bite; bite through clothes, shoes, or other protection; amount of venom injected; condition of fangs; and duration for which snake clings to the victim, all affect outcome
Snake species	Different species have different lethal dose, lethal period, and aggressiveness
Secondary infection	Presence or absence of pathogenic organisms in the mouth of the snake
Treatment	Nature of first aid given and time elapsed before first dose of antivenom.

### Elapid bites

Bites by krait, coral snake, and some cobras are associated with minimal local changes; however, bite by the Indian cobra (Naja naja) results in tender local swelling, blistering, and necrosis. Local necrosis causes a picture of “wet gangrene” with a characteristic putrid smell due to the direct cytolytic action of the venom. “Skip lesions” are typical findings. Systemic absorption occurs through venous channels and result in neurotoxic symptoms. Nausea, vomiting, malaise, prostration, and abdominal pain are the usual initial systemic symptoms. Paralysis is heralded by ptosis, followed by ophthalmoplegia. Paralysis of facial, palatal, tongue, and neck muscles follow. Respiratory failure, precipitated by upper airway obstruction and paralysis of intercostals and diaphragm, is the usual cause of death.[3,9]

### Viper bites

Viper bite is primarily vasculotoxic. It causes rapidly developing swelling of the bitten part. Local necrosis is mainly ischemic as thrombosis blocks the local blood vessels and causes a dry gangrene. Systemic absorption is slow; it occurs via the lymphatics and leads to lymphangitis. Hemostatic abnormalities are characteristic of viper bites and are the cause of the complications that lead to death. A persistent ooze from the bite mark and the site of the IV cannula is an indication of the altered clotting mechanism. Hemorrhage and increased capillary permeability leads to shock and pulmonary edema. Oliguria ensues, followed by loin pain due to renal ischemia. Renal failure is the common event before death.[3,9]

### Sea snake bite

The effects of a sea snake bite are both myotoxic and neurotoxic and result in clinical and pathological changes typical of segmental myopathic lesions in the skeletal muscles. Muscle pains may last for several months unless treated. Myoglobin and potassium released from damaged skeletal muscle can cause renal failure, while the hyperkalemia thus produced may lead to cardiac arrest.[3]

The average fatal dose, LD50, in mice and the average time to fatality of various snakes poison is given in Table 3.

**Table 3:** Various snakebites, their fatal dose, quantity of venom injected, and time to fatality

Snake	LD_50_ in mine	Fatal dose for humans	Average delivered dose per bite	Average fatal period
Indian cobra *(Naja naja)*	0.28 mg/kg	12 mg	60 mg	8 h
Common krait *(Bungarus caeruleus)*	0.09 mg/kg	6 mg	20 mg	18 h
Russell's viper *(Daboia russelii)*	0.1 mg/kg	15 mg	63 mg	3 days
Saw-scaled viper *(Echis carinatus)*	6.65 mg/kg	8 mg	13–40 mg	41 days

## MANAGEMENT OF SNAKEBITE

WHO/SEARO has published guidelines, specific for the South East Asia region, for the clinical management of snakebites; these guidelines appeared in the supplementary issue of the South East Asian Journal of Tropical Medicine and Public Health.[14]

WHO/SEARO guidelines are universally followed. The following management is as per the WHO guidelines

### First aid

The aim of first aid is to retard the systemic absorption of venom and prevent life-threatening complications by prompt transport to a medical facility. First aid can be performed by victim himself/herself or by any person who happens to be nearby. Traditionally, first aid included making local incisions or “tattooing” at the site of the bite, attempts at suctioning venom out of the wound, use of tight bands (tourniquets) around the limb, and/or local application of ice packs. None of the traditional remedies have any proven medical benefit. They should be discouraged as they do more harm than good and delay transport to a medical facility. Incision, suction, electric shocks, cryotherapy, or washing the wound are contraindicated as any interference with the wound introduces infection, increases bleeding from the site, and hastens absorption of the venom.

The current guidelines for first aid include the following:

1. Reassure the victim (70% of all snakebites are by nonvenomous snakes and 50% of bites by venomous species are dry bites[19])
2. Immobilize the affected limb (by bandage or clothes to hold splint, but tight arterial compression is not recommended)
3. Promptly transfer of victim to hospital

Pressure immobilization method (PIM) was developed by the Australian Venom Research Unit, University of Melbourne, Australia, for rapidly acting neurotoxic elapid snake venom. As per the PIM, immobilization and bandaging of the bitten part is similar to that done in the case of a sprained ankle. Studies have shown that it is seldom applied correctly[20] in simulated environments and, moreover, mobilizing the limb for more than 10 min nullifies the benefits of even the correctly applied bandage.[21]

In most instances, health care providers, general public, or community health workers are the first responders to come to the aid of the snakebite victim. If outcomes are to be improved, it is vital that they should all be made aware of the importance of immediate immobilization of the limb and transfer to the hospital at the earliest.

### Hospital treatment

#### Emergency care department

When the patient reaches the emergency department, evaluation should begin with the assessment of the airway, breathing, circulatory status, and consciousness.

Urgent resuscitation will be needed in those in shock (cardiovascular toxicity), those with respiratory failure (neurotoxin), and in those who have had cardiac arrest (due to hypoxia, cardiac toxicity, or hyperkalemia from rhabdomyolysis).

Oxygen should be administered to every envenomed patient and a large-bore intravenous catheter should be inserted. A bolus of normal saline or Ringer's lactate should be given to all patients with suspected envenomation. The patient may then be administered specific treatment after a precise history has been taken and thorough physical examination done.

#### History

Attempts should be made to determine whether a venomous snake has actually bitten the patient and, if so, the severity of the bite [Table 4]. It is essential to establish that the victim has been bitten by a snake and not by some other animal; this can be cross-checked by looking for fang marks and signs of local envenomation. If the victim has brought the snake, identification of the species should be carried out carefully, since crotalids can envenomate even when dead. This is why bringing the killed snake into the ED should be discouraged. Questions should be asked to determine the time elapsed since the snakebite and a brief medical history should be obtained (e.g., date of last tetanus immunization, use of any medication, presence of any systemic disease, and history of allergy).[9]

**Table 4:** Assessment of severity of envenomation

No envenomation	Absence of local or systemic reactions; fang marks (+/−)
Mild envenomation	Fang marks (+), moderate pain, minimal local edema (0–15 ce), erythema (+), ecchymosis (+/−), no systemic reactions
Moderate envenomation	Fang marks (+), severe pain, moderate local edema (15–30 cm), erythema and ecchymosis (+), systemic weakness, sweating, syncope, nausea, vomiting, anemia, or thrombocytopenia
Severe envenomation	Fang marks (+), severe pain, severe local edema (>30 cm), erythema and ecchymosis (+), hypotension, paresthesia, coma, pulmonary edema, respiratory failure

#### Physical examination

During the initial evaluation, the bite site should be examined for signs of local envenomation (edema, petechiae, bullae, oozing from the wound, etc) and for the extent of swelling. The bite site and at least two other, more proximal, locations should be marked and the circumference of the bitten limb should be measured every 15 min thereafter, until the swelling is no longer progressing. The extremity should be placed in a well-padded splint for at least 24 h. Serial measurement of circumference helps in estimating spread of venom and effect of antivenom.[9] Lymph nodes draining the limb should be palpated and the presence of lymphangitic lines noted.

Distal pulses should be checked and monitored if there is presence of gross swelling. The presence of a pulse does not rule out compartment syndrome however, and compartment pressure should be measured directly if there is concern that a compartment syndrome is developing. The diagnosis is established if the compartment pressure, measured directly by inserting a 22G IV cannula and connecting it with manometer, is raised above 55 cm water/saline. Direct measurement is necessary before resorting to fasciotomy since compartment syndrome is rare in snakebite victims and fasciotomy done without correction of hemostatic abnormality may cause the patient to bleed to death.[14]

Clues for severe snake envenomation should be sought.[14] They consist of the following:

1. Snake identified is a very venomous one
2. Rapid early extension of local swelling from the site of the bite
3. Early tender enlargement of local lymph nodes, indicating spread of venom in the lymphatic system
4. Early systemic symptoms
5. Early spontaneous systemic bleeding (especially bleeding from the gums)
6. Passage of dark brown urine

## LABORATORY INVESTIGATIONS

Although lab tests are of little value in the diagnosis of snake envenomation, they are useful for prognosticating and for making decisions about specific interventions.[22]

### Specific investigations

**(a) The 20-min whole blood clotting test (20 WBCT):** The 20 WBCT is a simple bedside test of coagulopathy to diagnose viper envenomation and rule out elapid bite. It requires a new clean, dry test tube made up of simple glass that has not been washed with any detergent. A few milliliters of fresh venous blood is drawn and left undisturbed in the test tube for 20 min; the tube is then tilted gently. If the blood is still liquid after 20 min, it is evidence of coagulopathy and confirms that the patient has been bitten by a viper. Cobras or kraits do not cause antihemostatic symptoms.[23]

**(b) Enzyme linked immunosorbent assay (ELISA):** ELISA tests are now available to identify the species involved, based on antigens in the venom.[24] These tests, however, are expensive and not freely available and thus have limited value in diagnosis; at present, they find use mainly in epidemiological studies.

### Other nonspecific tests include

- Hemogram: The hemogram may show transient elevation of hemoglobin level due to hemoconcentration (because of the increased capillary leak) or may show anemia (due to hemolysis, especially in viper bites). Presence of neutrophilic leucocytosis signifies systemic absorption of venom.[14] Thrombocytopenia may be a feature of viper envenomation.
- Serum creatinine: This is necessary to rule out renal failure after viper and sea snake bite.
- Serum amylase and creatinine phosphokinase (CPK): Elevated levels of these markers suggests muscle damage (caution for renal damage).
- Prothrombin time (PT) and activated partial thromboplastin time (aPTT): Prolongation may be present in viper bite.
- Fibrinogen and fibrin degradation products (FDPs): Low fibrinogen with elevated FDP is present when venom interferes with the clotting mechanism.
- Arterial blood gas and electrolyte determinations: These test are necessary for patients with systemic symptoms.
- Urine examination: Can reveal hematuria, proteinuria, hemoglobinuria, or myoglobinuria. (Arterial blood gases and urine examination should be repeated at frequent intervals during the acute phase to assess progressive systemic toxicity).
- Electrocardiogram (ECG): Nonspecific ECG changes such as bradycardia and atrioventricular block with ST-T changes may be seen.[25]
- Electroencephalogram (EEG): Recently, EEG changes have been noted in up to 96% of patients bitten by snakes. These changes start within hours of the bite but are not associated with any features of encephalopathy. Sixty-two percent showed grade I changes, 31% cases manifested grade II changes (moderate to severe abnormality), and the remaining 4% showed severe abnormality (grade III). These abnormal EEG patterns were seen mainly in the temporal lobes.[26]

The first blood drawn from the patient should be typed and cross-matched, as the effects of both venom and antivenom can interfere with later cross-matching.

## SPECIFIC THERAPY

### Anti–snake venom

Anti–snake venom (ASV) are immunoglobulins prepared by immunizing horses with the venom of poisonous snakes and subsequently extracting and purifying the horses' serum. They are the only effective antidote for snake venom. Antivenoms may be species specific (monovalent/monospecific) or may be effective against several species (polyvalent/polyspecific). Antibodies raised against the venom of one species may have cross-neutralizing activity against other venoms, usually that from closely related species. This is known as paraspecific activity. As per the recommendations of WHO, the most effective treatment for snakebite is the administration of monospecific ASV[27]; however, this therapy is not always available to snakebite victims because of its high cost, frequent lack of availability, and the difficulty in correctly identifying the snake.[3]

WHO recommends that if an adequate cold chain is in place, antivenoms should be prepared in the liquid form, since this reduces production costs and avoids the potential adverse physicochemical alterations to the product sometimes brought about by lyophilization. On the other hand, if the integrity of the cold chain cannot be guaranteed, antivenoms should be lyophilized to maintain stability.[19]

Several antivenom preparations are available internationally. In India, polyvalent antivenom prepared by Central Research Institute, Kasauli (HP) is effective against the most common Indian species [Table 5]. Antivenom produced at the Haffkine Corporation, Parel (Mumbai) is effective against the venom of even more species. Table 6 lists ASV producers in India, both in the public as well as the private sector.

**Table 5:** Polyvalent anti–snake venom serum produced by Central Research Institute, Kasauli (Himachal Pradesh)

Species	Type of antibody
*Bungarus caeruleus*	Specific
*Bungarus fasciatus*	Specific
*Calliophis species*	Specific
*Daboia russelii russelii*	Specific
*Echis carinatus*	Specific
*Hemibungarus*	Paraspecific
*Naja naja*	Specific
*Ophiophagus hannah*	Paraspecific
*Trimeresurus*	Paraspecific

**Table 6:** Anti-snake venom producers in India

Public sector	Private sector
Central Research Institute (CRI), Shimla Hills, Kasauli, HP	Serum Institute of India Ltd. (SII), Pune
Haffkine Biopharmaceutical Company Ltd (HBPCL), Mumbai	VINS Bioproducts Ltd., Hyderabad
King's Institute of Preventive Medicine (KIPM), Chennai	Biological E Ltd., Hyderabad
Bengal Chemicals and Pharmaceuticals Ltd., Kolkata	Bharat Serum and Vaccine Ltd.

ASV is supplied in dry powder form and has to be reconstituted by diluting in 10 ml of normal saline/D_5_ W. Mixing is done by swirling and not by vigorous shaking.

### Indications for ASV

The correct use of antivenom is essential and requires an informed evaluation of the patient. Not every poisonous snakebite merits its use. Antivenom treatment carries a risk of severe adverse reactions and in most countries it is costly and may be in limited supply. It should therefore be used only in patients in whom the benefits of antivenom treatment are considered to exceed the risks. Crotalidae polyvalent immune Fab (ovine) (CroFab; FabAV) has recently been approved for use in the United States. CroFab is a venom-specific Fab fragment of immunoglobulin G (IgG) that works by binding and neutralizing venom toxins, facilitating their redistribution away from target tissues and their elimination from the body. It has been demonstrated that these fragments are safe and effective, with a low incidence of sequelae; however, allergic reactions can occur when any animal protein derivatives are administered to human subjects. The overall incidence of immediate and delayed allergic reactions to this product appears so far to be lower than that reported with conventional whole-IgG antivenom.[28] Antivenom is indicated whenever there are signs of systemic envenomation or presence of severe local swelling. See Table 7 for details.

**Table 7:** Indications for anti-snake venom

System	Clinical features
	Spontaneous systemic bleeding
	Whole blood clotting time >20 min
	Thrombocytopenia (platelets <100,000/mm3)
	Shock
	Arrhythmia
	Abnormal electrocardiogram
Neurological	Ptosis and paralysis
Renal	Acute renal failure
	Generalized rhabdomyolysis and muscular pains
	Hyperkalemia
	Local swelling involving more than half of the bitten limb
	Rapid extension of swelling
	Development of an enlarged lymph node draining the bitten limb

### Antivenom therapy

Antivenom should be ideally administered within 4 h of the bite, but is effective even if given within 24 h. The dosage required varies with the degree of envenomation.

### Dose of ASV

Despite widespread use of antivenom, there have been virtually no clinical trials to determine the ideal dose.[29] The dosage has remained a matter of much debate. The conventional dosing in our setup is based on the degree of envenomation [Table 8].

**Table 8:** Conventional dose of anti-snake venom

Degree of envenomation	Initial dose
Mild	5 vials (50 ml)
Moderate	5–10 vials (50–100 ml)
Severe	10–20 vials (100–200 ml)

Additional infusions containing 5–10 vials (50–100 ml) are repeated until progression of swelling in the bitten part ceases and systemic signs and symptoms disappear

WHO/SEARO recommends the dose of antivenom to be the amount required to neutralize the average venom yield when captive snakes are milked of their venom.[27] Published research has indicated that the Russell's viper injects, on average, 63 mg (SD: ± 7 mg) of venom in the first bite.[30,31] As each vial of polyvalent ASV neutralizes 6 mg of Russell's viper venom, the initial dose should be 8–10 vials to ensure that the majority of the victims are covered by the initial dose; this will also help keep the cost of ASV down to acceptable levels.[32] As snakes inject the same amount of venom into children and adults, children should receive the same dose of antivenom as adults.[21]

Response to infusion of antivenom is marked by normalization of blood pressure.[33] Within 15–30 min bleeding stops, though coagulation disturbances may take up to 6 h to normalize. Neurotoxicity begins to improve within the first 30 min, but patients may require 24–48 h for full recovery.[34]

A repeat dose of ASV should be given when there is persistence of blood incoagulability even after 6 h or continued bleeding after 1–2 h of the initial dose. ASV should also be repeated when there are worsening neurotoxic or cardiovascular signs even after 1–2 h.[14]

### ASV administration

ASV can be administered either by slow intravenous injection at a rate of 2 ml/min or by intravenous infusion (antivenom diluted in 5–10 ml per kilogram body weight of normal saline or D_5_ W and infused over 1 h). Slow intravenous injection has the advantage that a doctor or nurse is present during the injection period when there is a risk of some early reaction to the ASV. All patients should be strictly observed for an hour for development of any anaphylactic reaction. Epinephrine should always be kept ready before administration of antivenom.

ASV should never be given locally at the site of the snakebite since it has not been shown to be effective and, moreover, this route of administration is associated with significant risks. For example, it is extremely painful and may increase intracompartmental pressure. Intramuscular injections are also not preferred since ASV is composed of large molecules (IgG or fragments) which are absorbed slowly via lymphatics, making the bioavailability by this route poor as compared to intravenous administration. Other disadvantages include pain on injection and risk of hematoma formation and sciatic nerve damage in patients with hemostatic abnormalities.[14] Intramuscular injections should only be given in settings where intravenous access cannot be obtained and/or the victim cannot be transported to a hospital immediately.

### ASV sensitivity testing

ASV sensitivity testing is no longer recommended as a lack of response does not predict the large majority of early (anaphylactic) or late (serum sickness type) reactions.[14] Such testing could also presensitize the patient to the serum protein and, in addition, often delays treatment.

### ASV reaction

Approximately 20% patients treated with ASV develop either early or late reaction.[14]

**Early anaphylactic reactions** occurs within 10–180 min of start of therapy and is characterized by itching, urticaria, dry cough, nausea and vomiting, abdominal colic, diarrhea, tachycardia, and fever. Some patients may develop severe life-threatening anaphylaxis characterized by hypotension, bronchospasm, and angioedema.

**Pyrogenic reactions** usually develop 1–2 h after treatment. Symptoms include chills and rigors, fever, and hypotension. These reactions are caused by contamination of the ASV with pyrogens during the manufacturing process.

**Late (serum sickness–type) reactions** develop 1–12 (mean 7) days after treatment. Clinical features include fever, nausea, vomiting, diarrhea, itching, recurrent urticaria, arthralgia, myalgia, lymphadenopathy, immune complex nephritis and, rarely, encephalopathy.

### Treatment of ASV reaction

When the patient shows signs of a reaction, antivenom administration must be temporarily stopped and adrenaline (1 in 1000) given intramuscularly in an initial dose of 0.5 mg in adults or 0.01 mg/kg body weight in children. The dose can be repeated every 5–10 min if necessary.

After adrenaline, an anti-H1 antihistamine such as chlorpheniramine maleate (adult dose 10 mg, children 0.2 mg/kg) should be given intravenously. It may be followed by intravenous hydrocortisone (adult dose 100 mg, children 2 mg/kg).

Late (serum sickness–type) reactions usually respond to a 5-day course of oral antihistamine (e.g., chlorpheniramine 2 mg six hourly in adults and 0.25 mg/kg/day in divided doses in children). Patients who fail to respond within 24–48 h should be given a 5-day course of prednisolone (5 mg six hourly in adults and 0.7 mg/kg/day in divided doses in children).

## SUPPORTIVE THERAPY

The patient should be moved to an appropriate area of the hospital. The ICU will be required for patients with signs of severe envenomation (coma, respiratory paralysis, hypotension, pulmonary edema, and history of syncope). Patients with presence of fang marks, moderate pain, minimal local edema, erythema, ecchymosis, and no systemic reactions can be treated in the ward under close monitoring. Supportive therapy is required to buy time while the damaged organs recover. The types of supportive care that may be needed is summarized below.

### Coagulopathy with bleeding

Coagulopathy usually reverses after ASV treatment. In exceptional cases, when there is severe bleeding or when urgent surgery is necessary, restoration of coagulability can be accelerated by giving fresh frozen plasma, cryoprecipitate (fibrinogen, factor VIII), fresh whole blood, or platelet concentrates.[27]

### Neurotoxic symptoms

Antivenom treatment alone cannot be relied upon to save the life of a patient with bulbar and respiratory paralysis. Once there is loss of the gag reflex, failure to cough, or respiratory distress, endotracheal intubation and initiation of mechanical ventilation is indicated. Tracheostomy and placement of a cuffed tracheostomy tube can be done whenever expertise for endotracheal intubation is not available. Since Elapid toxin result in pathophysiological changes resembling those of myasthenia gravis, anticholinesterase drugs can have a useful effect in patients with neurotoxic envenomation, especially in those bitten by cobras. A trial of anticholinesterase should be performed in every patient with neurotoxic envenomation. Injection neostigmine can be given as 50–100 μg/kg 4 hourly or as a continuous infusion. Glycopyrrolate 0.2 mg can be given before neostigmine as, unlike atropine, glycopyrrolate does not cross the blood–brain barrier. Seneviratne and Dissanayake,[15] in a prospective study on the neurological manifestations, disease course, and outcome in neurotoxic envenomation, demonstrated that neostigmine improved the muscle weakness. However, the number of cases in the study was too small for them to make an unequivocal recommendation. They were of the opinion that it would perhaps be reasonable to offer anticholinesterase therapy to those who demonstrate a positive response to the tensilon test or a decremental response to repetitive nerve stimulation.[15]

### Care of bitten part

The appearance of an immobile, tensely-swollen, cold, and apparently pulseless snake-bitten limb may suggest to surgeons the possibility of increased intracompartmental pressure, especially if the digital pulp spaces or the anterior tibial compartment are involved. Swelling of envenomed muscle within such tight fascial compartments could result in an increase in tissue pressure above the venous pressure and result in ischemia. However, the classical signs of an intracompartmental pressure syndrome may be difficult to assess in snakebite victims. Fasciotomy should not be contemplated until hemostatic abnormalities have been corrected, otherwise the patient may bleed to death. It has also been reported that fasciotomy worsens the amount of myonecrosis in crotalid snake venom–injected tissue.[35]

As most snakes harbor aerobic as well as anaerobic bacteria in their mouths, a prophylactic course of penicillin (or erythromycin for penicillin-hypersensitive patients) and a single dose of broad spectrum antibiotic course which will cover anaerobes together with a booster dose of tetanus toxoid is recommended.[27]

## CONCLUSION

Snakes do not generally attack human beings unless provoked. However, once bitten, a wide spectrum of clinical manifestations may result. The emphasis should be on early and adequate medical management. Delayed medical management and lack of public awareness results in prolonged hospital and ICU stay of the patients. This can be decreased if regular public programmes regarding prevention, prehospital management (first aid), and the importance of early transfer to hospital are conducted.

Overemphasis on reducing the load of snake venom in the victim during prehospital management can be dangerous because its role is debatable and too much valuable time is wasted in its administration. Most of the traditional methods for first aid treatment of snakebite, both western and “traditional/herbal,” have been found to result in more harm than good. Identification of the species of snake responsible for the bite is important for optimal clinical management. Antivenom is the only effective antidote for snake venom. However, it is expensive and usually in short supply and its use carries the risk of potentially dangerous reactions.
